# Low-Power Electrochromic Displays Based on Electrocatalytic Counter Electrodes and PVDF-HFP Gel Polymer Electrolyte

**DOI:** 10.3390/ma19071364

**Published:** 2026-03-30

**Authors:** Liangliang Wu, Lili Liu, Fengchao Li, Qiang Li, Lingqi Wu

**Affiliations:** 1 State Key Laboratory of Widegap Semiconductor Optoelectronic Materials and Technologies, School of Semiconductor and Physics, North University of China, Taiyuan 030051, China; lililiu@nuc.edu.cn (L.L.); lifengchao@nuc.edu.cn (F.L.); 2 School of Instrument and Electronics, North University of China, Taiyuan 030051, China; liqiang@nuc.edu.cn; 3 State Key Laboratory for Mechanical Behavior of Materials, School of Material Science and Engineering, Xi’an Jiaotong University, Xi’an 710049, China; wlq13840384707@163.com

**Keywords:** electrochromic displays, electrocatalytic, gel polymer electrolyte

## Abstract

Electrochromic devices have emerged as promising candidates for non-emissive displays due to their particular photoelectric performance in complex lighting environments. They exhibit considerable potential in emerging fields such as Internet of Things terminals, flexible wearables and human–computer interaction interfaces. In this study, we developed a low-power electrochromic display based on a Pt/FTO (Fluorine doped tin oxide) electrocatalytic counter electrode and a Poly(vinylidene fluoride-co-hexafluoropropylene) (PVDF-HFP) porous gel electrolyte. The Pt catalyst enhances Br^−^/Br^3−^ redox reactivity, which reduces the driving voltage from 2 V to 1 V, and accelerates the electrode reaction kinetics. It is systematically explained by the Density Functional Theory (DFT) calculations and electrochemical characterization. Furthermore, we demonstrate a proof-of-concept multicolor display incorporating the electrocatalytic counter electrode with various viologen derivatives. This approach provides a significant advancement toward next-generation high-performance displays and is supportive of the development of energy-efficient optoelectronic devices.

## 1. Introduction

In the era of the rapid advancement of Artificial Intelligence and Internet of Things technology, displays have emerged as pivotal gateways in human–computer interaction [1,2,3]. Emissive displays, represented by Light Emitting Diode (LED) and organic light-emitting diode (OLED) devices, are currently the dominant display technologies. These displays generate images via self-emissive light, and exhibit excellent display quality and high refresh rates [4,5]. However, their visibility decreases under bright ambient light due to glare from strong reflections. In recent years, electrochromism offers an alternative display technology that produces visual images through electrochemically induced redox reactions [6,7,8]. Such displays effectively avoid the harmful radiation of intense blue light and maintain optimal visual legibility in bright ambient conditions [6]. Nevertheless, electrochromic displays face significant challenges in their development, including stringent energy consumption requirements and insufficient display precision [8,9]. Therefore, it is highly desired to develop an electrochromic display with lower driving voltage and optimized display performance to facilitate its broader application in various fields.

In general, an electrochromic display is composed of active materials, an electrolyte, and two electrodes [10]. Among these components, catalytic materials are typically employed as the counter electrode to enhance the electrochromic performance [11]. For instance, Xu et al. [12] utilized a NiO-Pt counter electrode to fabricate high-performance electrochromic devices with a TMTU/TMFDS^2+^ redox couple. Ma et al. [13] developed fast-switching electrochromic smart windows based on NiO-nanorods counter electrodes. Fang et al. [14] proposed a high performance large-area viologen-based electrochromic device with a Pt/ITO (Indium Tin Oxide) counter electrode. These results indicate that the electrode reaction potential in electrochemical devices is influenced by the electrocatalytic activity of the electrode [15]. Thus, it is a predictable method to enable a low-power information display utilizing a patterned catalytic counter electrode. In addition, viologen is an organic molecular electrochromic compound with three redox states. The large difference in molar extinction coefficient between its dicationic (V^2+^) and radical cationic (V^+^) states results in distinct macroscopic color changes. Furthermore, the modulation of electronic orbital energy levels by substituents endows viologens with outstanding color tunability. Accordingly, viologen represents a promising candidate for multicolor electrochromic display devices. However, the universality of the catalytic counter across various viologen derivatives remains insufficiently explored up to now.

Electrolytes serve as the primary medium for ionic conduction in electrochromic devices, directly influencing the response time and operating voltage [16]. In particular, polymer gel electrolytes, composed of polymer networks and electrolyte solutions, have been widely used in electrochromic devices due to their high ionic conductivity and excellent solution processability [17,18,19]. For electrochromic displays, the thickness of the electrolyte layer must be taken into consideration to mitigate signal crosstalk during lateral ion transport [20]. A thin electrolyte layer is conducive to achieving rapid response and enhancing display resolution. Currently, poly(vinylidene fluoride-co-hexafluoropropylene) (PVDF-HFP) film can be prepared via film casting and extraction processes, which enable precise control over film thickness [21,22]. In addition, PVDF-HFP film has been proven to be an effective ion gel electrolyte in electrochromic devices due to its high mechanical strength from the crystalline VDF units and the ability to trap electrolytes in the amorphous phase of HFP units [23,24,25]. Thus, the PVDF-HFP film serves as promising candidate for the electrolyte in high-precision electrochromic displays.

In this work, we demonstrate an electrochromic display based on a Pt catalytic counter electrode and a PVDF-HFP porous membrane polymer electrolyte. The as-prepared electrochromic display exhibited comprehensive properties: lower driving voltage, enhanced spectral modulation and a faster response time. In addition, the charge transfer process on the Pt catalytic electrode was also verified through theoretical analysis and experimental validation. It is anticipated that the novel exploratory approaches and design principles for electrocatalytic counter electrodes will provide unique insights into various energy-saving and sustainable optoelectronic applications.

## 2. Materials and Methods

### 2.1. Material Preparation and Software Usage

PVDF-HFP powders were obtained from Sigma-Aldrich, St. Louis, MO, USA. Polyvinyl pyrrolidone (PVP), N,N-dimethylformamide, acetonitrile, and Pt particles were purchased from Shanghai Lanji Technology Development Co., Ltd. (Shanghai, China), Tianjin Tianli Chemical Reagents Ltd. (Tianjin, China), Shanghai Maclin Biochemical Technology Co., Ltd. (Shanghai, China), and China New Metal Materials Technology Co., Ltd. (Beijing, China), respectively. Acetone, methanol, and diethyl ether were supplied by Sinopharm Chemical Reagent Co., Ltd., Shanghai, China. 4,4′-Bipyridine and heptyl bromide were acquired from TCI Shanghai, Shanghai, China. Ethyl viologen (EV), propylene carbonate, phenyl viologen (PV), and fluorine-doped tin oxide (FTO) glasses were sourced from Shanghai Aladdin Bio-Chem Technology Co., Ltd. (Shanghai, China), Beijing Mreda Technology Co., Ltd. (Beijing, China), and Liaoning Youxuan New Energy Technology Co., Ltd. (Yingkou, China), respectively. DFT calculations were performed using the Vienna Ab initio Simulation Package (VASP). Electric field strength simulations were performed using COMSOL Multiphysics Version 5.6.

### 2.2. Preparation of PVDF-HFP Film

The PVDF-HFP film was synthetized by the solution casting method. The 20 wt% PVDF-HFP powders and 15 wt% PVP were dissolved in a mixed solution of N-N dimethylformamide and acetone with mass ratio of 1:1. The mixed solution was stirred at 30 °C, then cast onto a PTFE substrate using laboratory casting equipment (MSK-AFA-L800, Hefei Kejing Materials Technology Co., Ltd. (Heifei, China)) and completely dried overnight. Subsequently, the films were peeled from the PTFE substrate and soaked in methanol to remove the PVP. Finally, a porous PVDF-HFP film was obtained after drying.

### 2.3. Preparation of Monoheptyl Viologen

The monoheptyl viologen (MHV) was synthetized using 4,4′-bipyridine and heptyl bromide. First, 12 mmol 4,4′-bipyridine and 10 mmol heptyl bromide were dissolved in 20 mL acetonitrile. The resulting mixture was stirred at 50 °C for 72 h. Subsequently, the MHV product was collected via filtration, rinsed with diethyl ether and dried in a vacuum oven at 80 °C for 24 h.

### 2.4. Fabrication of the Electrochromic Device

The Pt film was prepared by vacuum evaporation on FTO electrode. The patterned Pt film was prepared using a mask during the vacuum evaporation. Additionally, ethyl viologen dibromide, phenyl viologen dichloride, and monoheptyl viologen dibromide were separately dissolved in propylene carbonate at a concentration of 5 mg/mL to prepare the electrolyte solutions. As viologen cations and halogen anions serve as conductive ions, no additional salt species were introduced into the electrolyte. Then, the porous PVDF-HFP film was completely immersed in electrolyte solution to obtain the film electrolyte. Finally, the electrochromic devices and multicolor display devices were fabricated by sandwiching the film electrolyte between the patterned Pt/FTO electrode and the bare FTO electrode.

### 2.5. Characterization

Field Emission Scanning Electron Microscopy (GeminiSEM 500, Zeiss, Oberkochen, Germany), Fourier Transform Infrared Spectroscopy (VERTEX70, BRUKER, Karlsruhe, Germany), and X-ray Diffractometry (D8 ADVANCE, BRUKER, Karlsruhe, Germany) were employed to characterize the as-prepared PVDF-HFP films. Atomic Force Microscopy (Dimension ICON, BRUKER, Karlsruhe, Germany) was used to examine the surface morphology and thickness of the Pt electrode. The chemical structure of synthesized monoheptyl viologen was verified via Nuclear Magnetic Resonance Spectroscopy (JNM-ECZ400S/L1, JEOL, Tokyo, Japan). Transmittance measurements of the electrochromic devices were performed using a UV-Vis spectrometer (V-1600PC, Shanghai Mepeda Instrument Co., Ltd., Shanghai, China), while a Digital SourceMeter (KEITHLEY 2410, Keithley, Solon, OH, USA) was utilized for current testing. Cyclic voltammetry (CV) and electrochemical impedance spectroscopy (EIS) were characterized with an electrochemical workstation (Zennium Pro, Zahner, Kronach, Germany).

## 3. Results and Discussion

**Figure 1:**
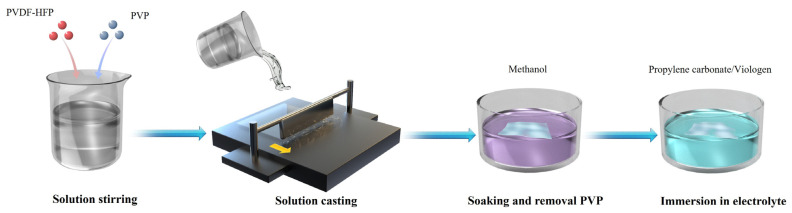
Schematic illustration of the preparation process of porous PVDF-HFP film.

**Figure 2:**
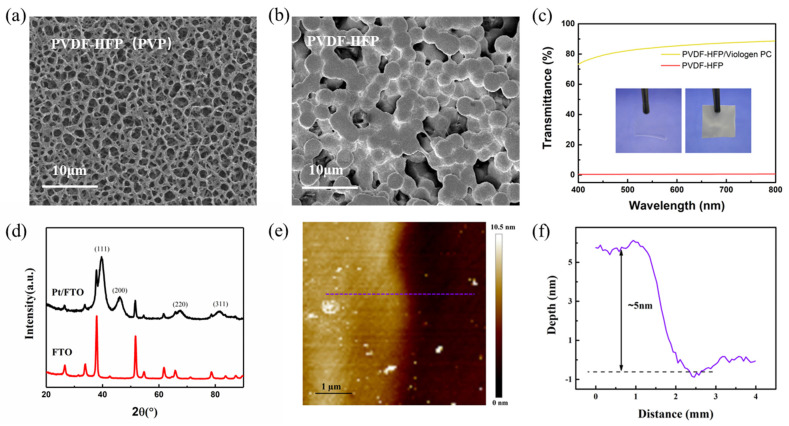
(**a**) SEM micrographs of the PVDF-HFP film with 15 wt% PVP, (**b**) SEM micrographs of the neat PVDF-HFP films, (**c**) Optical transmittance spectra of the porous PVDF-HFP film before and after immersion in electrolyte (inset are the micrographs of the porous PVDF-HFP film before and after immersion in electrolyte), (**d**) XRD patterns of the Pt/FTO and bare FTO, (**e**) AFM image of the surface morphology of the Pt on sapphire substrate, and (**f**) Change of surface layer thickness along the dotted line in figure (**e**).

The PVDF-HFP film was fabricated by the solution casting method as shown in Figure 1. Since the morphology and structure of electrolyte films are crucial for ion transport, PVP was added as a pore-forming agent. This addition enhances the electrolyte uptake capacity of the PVDF-HFP film. Compared to the PVDF-HFP film without PVP, micron-scale holes are uniformly distributed in the PVDF-HFP film with PVP as shown in Figure 2a,b. In addition, the composition and structure of the PVDF-HFP film were further analyzed by Fourier Transform Infrared (FTIR) spectrometry, as shown in Figure S1 (Supporting Information). Characteristic peaks located at 825 cm^−1^ (CF_3_ group), 875 cm^−1^ (vinylidene group), 1169 cm^−1^ (CF_3_ group), and 1394 cm^−1^ (CH=CF skeleton) confirmed the existence of a PVDF-HFP copolymer. In addition, two characteristic peaks were detected at 1295 cm^−1^ and 1667 cm^−1^, corresponding to the -C-N- stretching vibration and -C=O vibrational transition of residual PVP [26]. Figure 2c shows the transmittance of the porous PVDF-HFP(PVP) film before and after immersion in the viologen/propylene carbonate electrolyte. The transmittance is nearly 0% for the dry porous PVDF-HFP(PVP) film from 400 nm to 800 nm, which is due to the light scattering at the interface between the rough film and air. In contrast, the transmittance of the infiltrated PVDF-HFP(PVP) film is nearly 80% from 400 nm to 800 nm. This difference is attributed to the close refractive index between the propylene carbonate (1.422) solution and PVDF-HFP (1.420) film.

We further conducted a comprehensive characterization of the Pt/FTO counter electrode. As shown in Figure 2d, the XRD pattern exhibits characteristic diffraction peaks located at 39.7°, 46.2°, 67.5°, and 81.4°, which are assigned to the (111), (200), (220), and (311) crystal planes of cubic Pt nanoparticles (JCPDS No. 00-033-0875), thus verifying the successful deposition of Pt on the FTO substrate [27]. In addition, Atomic Force Microscopy (AFM) was employed to characterize the surface morphology and thickness of the Pt film deposited on a sapphire substrate. The AFM results presented in Figure 2e,f reveal that the Pt film has a uniform thickness of approximately 5 nm and a relatively smooth surface; such a favorable morphological feature guarantees its applicability in the fabrication of electrochromic devices.

**Figure 3:**
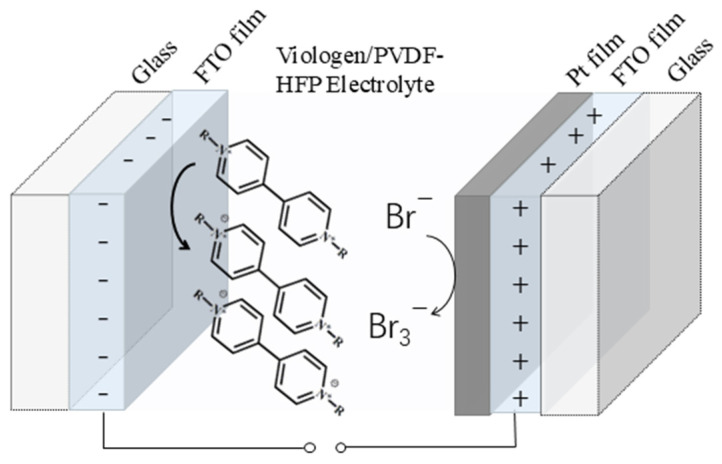
The schematic diagram of the electrochromic devices with a Pt/FTO counter electrode.

**Figure 4:**
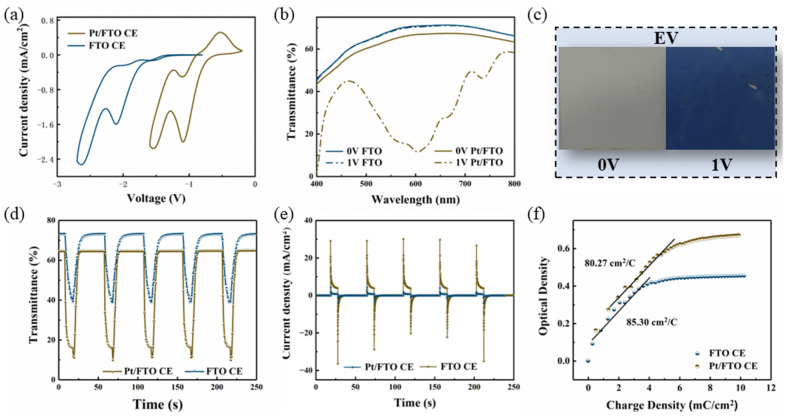
(**a**) Cyclic voltammetry (CV) profiles with FTO counter electrode and Pt/FTO counter electrode, (**b**) the transmittance curves of the device in 0 V and 1 V were measured at 0 V and 1 V, respectively, (**c**) photographs of the bleached and colored devices. (**d**) transmittance–time curves under voltage of 2 V for 10 s and 0 V for 40 s at 600 nm, (**e**) the chronoamperometric curve of the devices with FTO counter electrode and Pt/FTO counter electrode under pulse voltage of 2 V for 10 s and 0 V for 40 s, and (**f**) plots of the optical density versus injected charge density for electrochromic devices with the FTO counter electrode and the Pt/FTO counter electrode, respectively.

The electrochromic devices were fabricated by sandwiching the viologen/PVDF-HFP film electrolyte between Pt/FTO counter electrode and bare FTO work electrode, as shown in Figure 3. The electrochemical behavior of ethyl viologen (EV)-based electrochromic devices employing Pt/FTO and bare FTO counter electrodes was evaluated via an electrochemical workstation. Cyclic voltammetry (CV) curves of these two-electrode configurations are presented in Figure 4a. Two distinct reduction peaks are observed, corresponding to the sequential two-step redox reaction of viologen species during the coloring process [28,29]. As the color transition of viologen molecules initiates at the first reduction step, the potential of this first peak is defined as the coloring voltage of the viologen-based electrochromic device [30,31,32]. The first reduction peak of the Pt/FTO-counter-electrode device appears at 1 V, a notable decrease compared to the 2 V peak observed for the device with bare FTO counter electrode. In parallel, the transmittance spectra of both devices were recorded across 400–800 nm using a UV-Vis spectrometer, as displayed in Figure 4b. At 0 V, both devices remain in the bleached state. The bleached transmittance of the Pt/FTO-based device is lower than that of the control device, which can be attributed to light absorption by Pt nanoparticles decorated on the FTO surface. Upon applying 1 V, the transmittance of the Pt/FTO device drops sharply, whereas the transmittance of the FTO-only device remains nearly unchanged. This result confirms that the Pt/FTO device operates at a lower driving voltage, which aligns well with the lower coloring voltage observed in the CV curves of Figure 4a. Furthermore, the Pt/FTO device delivers a pronounced transmittance modulation (ΔT) of 54.8% at 600 nm under 1 V, and the optical photographs of the bleached and colored states (Figure 4c) visually validate this strong modulation performance.

Time-dependent transmittance responses and chronoamperometry profiles were recorded to probe the dynamic electrochromic switching behavior of the devices. As depicted in Figure 4d, the optical contrast (ΔT) of the Pt/FTO-counter-electrode device is nearly double that of the FTO-only device under dynamic pulse testing. Furthermore, the bleaching time of the Pt/FTO device (11 s) is markedly shortened relative to the control device (35 s), demonstrating faster optical recovery. The cycling stability of the Pt/FTO device is presented in Figure S2, confirming its potential for reliable long-term operation. A comparison of key electrochromic metrics with recently reported low-voltage devices is summarized in Table S1 (Supporting Information) [33,34,35,36,37]. The longer switching time compared to other gel electrolyte systems arises mainly from the larger size of viologen ions and the denser cross-linked gel network of PVDF-HFP. It indicates that this electrochromic device is suitable for low-frequency switching and static display applications, where slow response is acceptable and long-term durability is prioritized. Additionally, current-time responses under pulsed voltages (2 V for 10 s, 0 V for 40 s) are displayed in Figure 4e. The current density in the Pt/FTO device is notably higher than that in the FTO device, which stems from more efficient redox kinetics at the electrolyte–electrode interface. This finding aligns well with the enhanced optical modulation capability of the Pt/FTO device observed under identical testing conditions in Figure 4d.

Furthermore, coloration efficiency (CE) is an important indicator for evaluating the performance of the electrochromic device, which is defined as follows:

(1) $$\;CE=\frac{△OD}{△Q}=\frac{log(T_{b}/T_{c})}{△Q}$$

Here, Δ*OD* represents the change in optical density, defined as the common logarithm of the ratio between the transmittance in the bleached state (*Tb*) and the colored state (*Tc*). Δ*Q* denotes the variation in injected charge density (per unit area) corresponding to the measured Δ*OD*. The coloration efficiency values for devices with Pt/FTO and FTO counter electrodes are plotted in Figure 4f. The similar coloration efficiency indicates that the electrochromic reaction is consistent in both devices and Pt nanoparticles only act as a catalyst without altering the electrode reaction.

**Figure 5:**
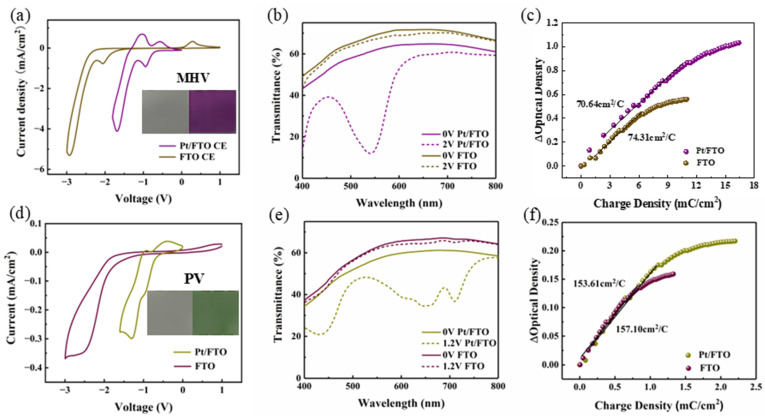
(**a**) Cyclic voltammetry (CV) profiles of the monoheptyl viologen (MHV) device in two-electrode system with FTO counter electrode and Pt/FTO counter electrode (inset: digital photographss of the bleached and colored MHV devices), (**b**) optical transmittance spectra of the MHV device with FTO counter electrode and Pt/FTO counter electrode, respectively, (**c**) plots of optical density (OD) versus injected charge density for MHV electrochromic devices with FTO counter electrode and Pt/FTO counter electrode, respectively, (**d**) CV profiles of the phenyl viologen (PV) device in two-electrode system with FTO counter electrode and Pt/FTO counter electrode (inset: photographs of the bleached and colored PV devices), (**e**) optical transmittance spectra of the PV device with FTO counter electrode and Pt/FTO counter electrode, respectively, and (**f**) OD versus charge density plots for PV electrochromic devices with FTO counter electrode and Pt/FTO counter electrode, respectively.

A broader spectrum of colors Is critical for practical applications of electrochromic displays. Viologen with different substituents exhibits distinct spectral absorption characteristics, which is due to the effect of substituents on the orbital energy levels of electrons [38]. To verify the universality of Pt/FTO electrocatalytic electrode for viologen with different substituents, we fabricated electrochromic devices using monoheptyl viologen (MHV) (purple) and phenyl viologen (PV) (green). Among them, monoheptyl viologen was synthesized via the solution method described in Section 2.3. To confirm the chemical composition of the synthetic monoheptyl viologen, a 1H NMR spectrum of the compound dissolved in DMSO-d6 was performed. As shown in Figure S3, peaks for the proton on the pyridine ring appear at around 8.06–9.31 ppm, and peaks for the proton on the alkyl are observed at around 0.83–4.67 ppm. Through integral analysis, there are 8 protons on the pyridine ring and 15 protons on the alkyl, which is consistent with the chemical formula of the monoheptyl viologen [39]. As shown in Figure 5a,d, the voltage corresponding to the first reduction peak decreases significantly in both electrochromic devices with the Pt/FTO counter electrode. It indicates that Pt/FTO counter electrode reduces the coloring voltage and this effect extends to various viologen derivatives. Moreover, these electrochromic devices also possess enhanced spectral modulation performance (Figure 5b,e). The dynamic characteristics of these electrochromic devices were then investigated. In Figure 5c,f, the coloration efficiency is close in the electrochromic devices using the same viologen derivative. The Pt/FTO counter electrode only acts as a catalyst, which is consistent with the results in Figure 4f. In general, the electrocatalysis of Pt/FTO is universal for various viologen-based electrochromic devices and holds great potential for multicolored electrochromic devices.

Within the electrochromic assembly, the reduction of viologen dications (V^2+^) at the working electrode occurs synchronously with the oxidation of bromide ions (Br^−^) at the counter electrode. The corresponding redox reaction at the Pt/FTO counter electrode during the coloring step is expressed as follows,

(2) $$3{Br}^{-}-2e^{-}↔{{Br}_{3}}^{-}$$

**Figure 6:**
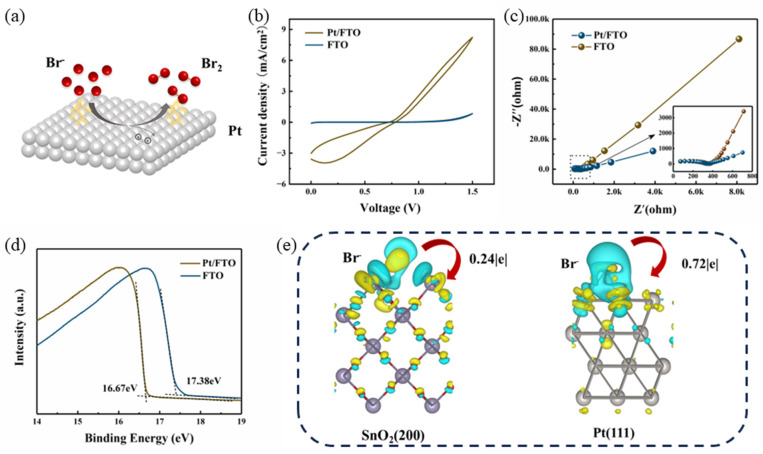
(**a**) Schematic illustration of the redox reactions occurring at the working and counter electrodes, (**b**) Cyclic voltammetry (CV) profiles of the three-electrode system with Pt/FTO and bare FTO as the working electrode, a Pt wire as the counter electrode, and Ag/AgCl (saturated KCl) as the reference electrode, (**c**) Nyquist plots of the aforementioned three-electrode system tested over a frequency range of 0.1 Hz to 4 MHz (inset: magnified plot for 3 Hz to 4 MHz), (**d**) Ultraviolet photoelectron spectroscopy (UPS) spectra of the bare FTO and Pt/FTO electrodes, and (**e**) calculated charge density difference maps for the Pt (111) and SnO_2_ (200).

The schematic illustration of the electrochemical reaction at the counter electrode is presented in Figure 6a. To quantitatively assess the electrocatalytic activity of the Pt-modified FTO counter electrode, the redox behavior of Br^−^ at the Pt/FTO interface was systematically investigated via cyclic voltammetry (CV) and electrochemical impedance spectroscopy (EIS) in a three-electrode configuration. As displayed in Figure 6b, the current on the Pt/FTO electrode starts to rise at a lower potential compared to the bare FTO electrode, confirming that the oxidation of Br^−^ is more thermodynamically favorable on the Pt/FTO surface. Meanwhile, the corresponding Nyquist plots of EIS are shown in Figure 6c. In this system, the semicircle in the high-frequency region corresponds to the charge-transfer process at the counter electrode–electrolyte interface, while the low-frequency semicircle reflects the charge-transfer kinetics at the working electrode–electrolyte interface. The smaller semicircle diameter of the Pt/FTO electrode in the high-frequency region reveals a lower charge-transfer resistance at the Pt/FTO/electrolyte interface, which aligns well with the enhanced Br^−^ oxidation activity observed in the CV results. Furthermore, the work functions of the Pt/FTO and FTO electrodes were measured by ultraviolet photoelectron spectroscopy (UPS), as depicted in Figure 6d. The work function of bare FTO is 3.84 eV (derived from 21.22 eV–17.3 eV), while that of Pt/FTO is 4.55 eV (derived from 21.22 eV–16.67 eV). This increase arises from the stronger electron-withdrawing ability of Pt, which is proposed to be the underlying mechanism for the reduced driving voltage of the electrochromic devices.

In addition, the charge transfer process is further analyzed by the density functional theory (DFT) to verify the catalytic performance of the Pt/FTO counter electrode. DFT calculations were performed using the Vienna Ab initio Simulation Package (VASP) based on density functional theory (DFT), with the exchange-correlation potential described by the Perdew–Burke–Ernzerhof (PBE) functional within the generalized gradient approximation (GGA). The electron–ion interactions were described by the projector-augmented wave (PAW). For the valence electrons, a plane-wave basis set was adopted with a 500 eV kinetic energy cutoff. For structural relaxation, the convergence threshold for atomic forces was 0.01 eV/Å and the total energy convergence threshold was 1 × 10^−5^ eV per atom; a Monkhorst–Pack k-point grid of 3 × 3 × 1 was used for Brillouin zone sampling. The Bader charge analysis was employed to quantitatively calculate the charge transfer between different atoms in the system. In Figure 6e, an electron-depleted region (green) appears around Br^−^, while there is an electron-accumulated region (blue) around the FTO (SnO_2_) (200) and Pt (111) surfaces, indicating that electrons are transferred from Br^−^ to the electrode surface. Calculated charge density difference results for the two systems reveal a much stronger electronic interaction between adsorbed Br^−^ anions and the Pt/FTO surface relative to the bare FTO electrode. These theoretical findings are in good agreement with our experimental data, confirming that the Pt/FTO electrocatalyst enhances the overall electrochromic performance of the device by accelerating interfacial reaction kinetics at the electrolyte/counter electrode boundary.

**Figure 7:**
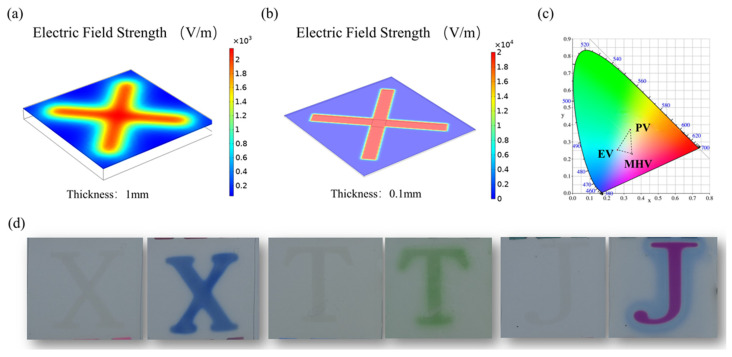
(**a**) Electric field strength in film electrolyte with a thickness of 1 mm, (**b**) Electric field strength in film electrolyte with a thickness of 0.1 mm, (**c**) color coordinate curves of three viologen-based electrochromic devices in CIE diagram and (**d**) photographs of the multicolored information display devices.

Next, we developed a multicolored electrochromic information display device to validate the practicality of the Pt/FTO counter electrode. The reduction reaction of viologen dication tends to occur on the working electrode corresponding to the Pt modified FTO counter electrode. Therefore, a Pt partially modified FTO can be used as a counter electrode to realize information display. When the electrolyte layer is thick, the accuracy of the information display will be reduced due to the transverse divergence of the electric field and lateral ion migration. In addition, the distribution of electric field strength between opposite electrodes with different spacing was simulated by COMSOL Multiphysics 5.6 using the AC/DC Module. The corresponding steady-state governing equations and key simulation parameters are detailed in Supporting Information (Table S2). As shown in in Figure 7a,b, COMSOL Multiphysics simulations demonstrate that reducing the thickness of the electrolyte film effectively mitigates transverse electric field divergence. Therefore, the PVDF-HFP electrolyte thin film in this work possesses certain advantages in high-precision multicolored electrochromic information display. Subsequently, we introduce blue ethyl viologen, green phenyl viologen, and purple monoheptyl viologen into electrochromic devices. Chromatic coordinates serve as a key characterization parameter for multicolor electrochromic devices, and the chromatic coordinate of these three viologen electrochromic information display devices can be calculated as follows [40]:

(3) $$\;\left[\begin{array}{c} X\\ Y\\ Z \end{array}\right]=\left[\begin{array}{ccc} 2.7689 & 1.7517 & 1.1302\\ 1.0000 & 4.5907 & 0.0601\\ 0.0000 & 0.0565 & 5.5943 \end{array}\right]\left[\begin{array}{c} R\\ G\\ B \end{array}\right]$$

(4) $$\left\{\begin{array}{c} x=\frac{X}{X+Y+Z}\\ y=\frac{Y}{X+Y+Z}\\ z=\frac{Z}{X+Y+Z} \end{array}\right.$$

Figure 7c shows the color coordinates of three viologen electrochromic information display devices in the CIE diagram. The colors in the area bounded by the three-color coordinates can theoretically be obtained by superimposing three viologen-based electrochromic devices. The photographs of the multicolored information display devices with an “XTJ” patterned Pt/FTO counter electrode are shown in Figure 7d. These devices can display obvious tricolored “XTJ” letters when driven by a low voltage. This work establishes a novel application paradigm for Pt/FTO counter electrodes in electrochromic systems and endows them with considerable potential for multicolor information display applications.

## 4. Conclusions

In summary, we have successfully developed a low-power multicolor electrochromic display utilizing a Pt/FTO electrocatalytic counter electrode. Pt nanoparticles exhibit excellent electrocatalytic activity toward the Br^−^/Br_3_^−^ redox couple, which markedly enhances the kinetics of the electrochemical reaction during the coloring process. The resulting electrochromic device delivers far superior performance relative to the control device with a bare FTO counter electrode, with the operating voltage reduced from 2 V to 1 V. The universality of the Pt/FTO electrocatalytic counter electrode has been verified in blue, green, and purple viologen electrochromic devices. Moreover, the enhanced charge transfer process is further theoretically explained by the DFT. A multicolored electrochromic display device was demonstrated by combining the multicolored viologen species and a PVDF-HFP electrolyte film, which provides a potential for the application of Pt/FTO electrocatalytic counter electrode for multicolored displays in low-frequency switching scenarios such as public information signage and electronic labels.

## Data Availability

The original contributions presented in this study are included in the article/Supplementary Materials. Further inquiries can be directed to the corresponding author.

## Supplementary Materials

The following supporting information can be downloaded at: https://www.mdpi.com/article/10.3390/ma19071364/s1, Figure S1: the FTIR of the PVDF-HFP film with PVP and PVDF-HFP film without PVP. Figure S2: The cycling stability of the device with Pt/FTO counter electrode for 100 cycles. Figure S3: 1H-NMR of the Monoheptyl Viologen. Table S1: The key metrics of the electrochromic device was compared with recent low-voltage electrochromic. Table S2: The key simulation parameters for COMSOL Multiphysics simulation.
