# Supplementary material for: Low-Power Electrochromic Displays Based on Electrocatalytic Counter Electrodes and PVDF-HFP Gel Polymer Electrolyte
Source: Materials (Basel). 2026 Mar 30;19(7):1364. doi: 10.3390/ma19071364 (PMC13074594; doi:10.3390/ma19071364)
Supplement: Supplementary file 1 [file materials-19-01364-s001.zip › materials-4178258-supplementary.pdf]

Supporting Information

Low-Power Electrochromic Displays Based on Electrocatalytic Counter Electrodes and PVDF-HFP Gel Polymer Electrolyte

Liangliang Wu, Lili Liu, Fengchao Li, Qiang Li, and Lingqi Wu

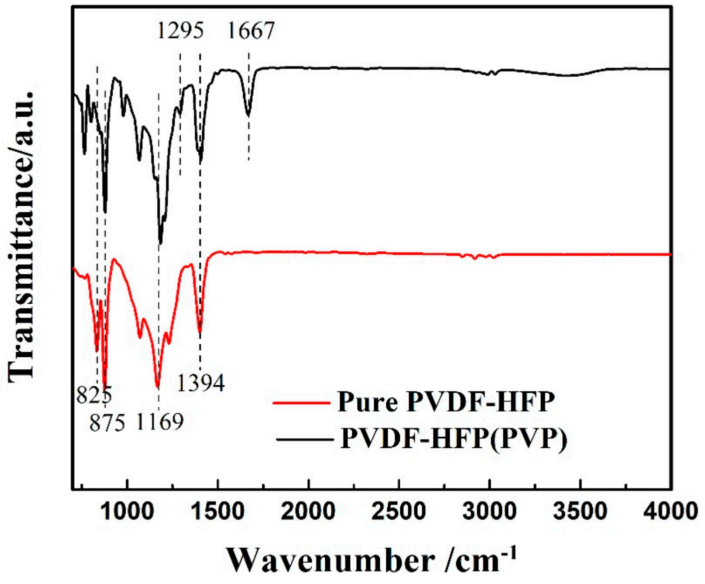

Figure.S1 the FTIR of the PVDF-HFP film with PVP and the PVDF-HFP film without PVP.

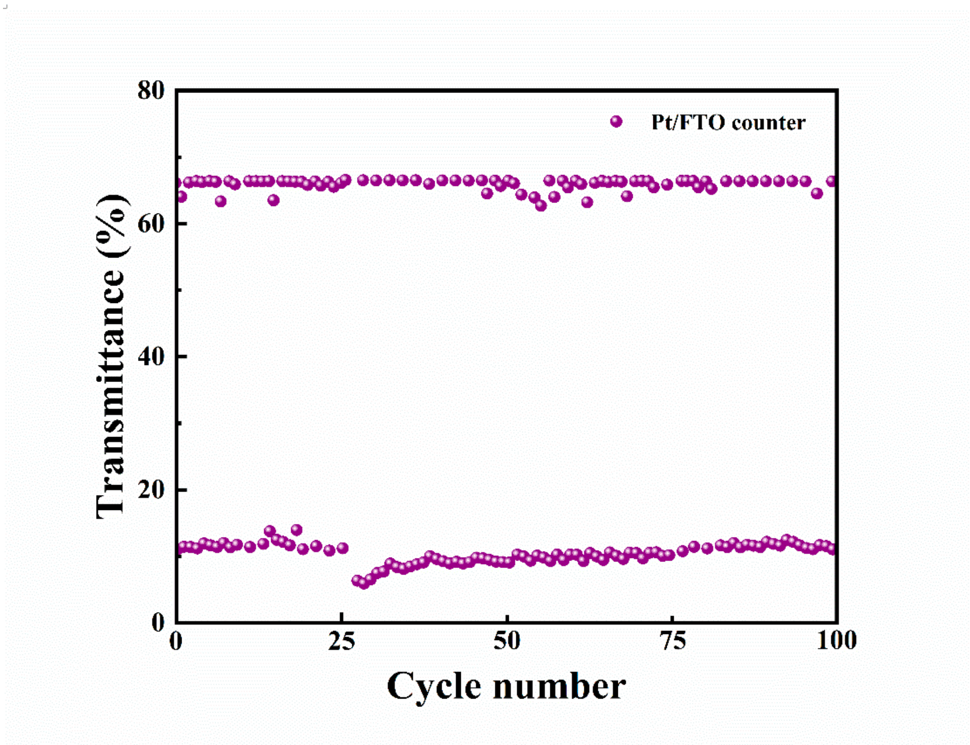

Figure.S2 The cycling stability of the device with Pt/FTO counter electrode for 100 cycles

Table. S1 The key metrics of the electrochromic device ~~was~~ were compared with recent low-voltage electrochromics

Reference	Device Type	Driving Voltage (V)	optical contrast,	Switching Time (s)
This work	FTO/viologen/PC/PVDF-HFP/Pt/FTO	1 V (coloring), 0 V (bleaching)	54.9% @ 600nm	10 s (coloring time), 11 s (bleaching time)
Ref. [1]	PET/PEDOT:PSS/Gel-electrolyte/PANI:PSS/PEDOT:PSS/PET	-1.8 (coloring), +0.4 (bleaching)	52.2% @ 600 nm	17.6 (coloring), 28.5 (bleaching)

Ref. [2]	ITO/CMC-Na-PAM double network hydrogel/BPYDB-C_6H_{13}	2.1V(coloring), 0 (bleaching)	30.3% @ 523 nm	7.4 (coloring), 5.0 (bleaching)
Ref. [3]	ITO/PANI/P3HT/Li-ion gel electrolyte	+0.5V(coloring)	68% @ 550 nm	0.5 (coloring), 0.5 (bleaching)
Ref. [4]	ITO/V_2O_5/TiO_2 gel/V_2O_5/ITO	+1V(coloring), -0.2(bleaching)	51.24% @ 800 nm	2s（coloring）
Ref. [5]	ITO/Fe (II)-MEPE/GET-BQ/ITO	~1.1(coloring), -0.5(bleaching)	35.7% @ 580 nm	0.6(coloring),4.2（bleaching）

Reference

[1] Park C.; Kim J. M.; Kim Y.; Bae S.; Do M.; Im S.; Yoo S. High-Coloration Efficiency and Low-Power Consumption Electrochromic Film based on Multifunctional Conducting Polymer for Large Scale Smart Windows and Jung Hyun Kim*High-Coloration Efficiency and Low-Power Consumption Electrochromic Film based on Multifunctional Conducting Polymer for Large Scale Smart Windows. *ACS Appl. Electron. Mater*. **2021**, 3, 4781–4792. https://doi.org/10.1021/acsaelm.1c00664

[2] Pan M.; He Q.; Liu J.; Du K.; Gong C.; Tang Q. A double network hydrogel electrolyte-based electrochromic device for enhanced electrochromic performance and lower power consumption. *DYES PIGMENTS* **2023**, 212, 11126. https://doi.org/10.1016/j.dyepig.2023.111126

[3] Pathak D. K.; Ghosh T.; Kandpal S.; Rani C. All-polymer solid state electrochromic device for low voltage and fast modulation between primary colors. Opt. Mater. 2023, 137, 113519. https://doi.org/10.1016/j.optmat.2023.113519

[4] Shin D.; Kim J.; Choi S.; Song G.; Rougier A. Caroline Sunyong Lee Evaluation of low-voltage-driven multi-colored electrochromic device based on dry-deposited V_2O_5. *SOL. ENERGY MATER. SOL. CELLS* **2023**, 257, 112341. https://doi.org/10.1016/j.solmat.2023.112341

[5] Xu L.; Li Y.; Ji Y.; Zhang J.; Shao L.; Zhang D.; Mac L.; Gao G. Zaixing Jiang Low-power, fast response simplified electrochromic device based on functionalized gel electrolyte. *Chem. Eng. J.* **2023**, 467, 143400. https://doi.org/10.1016/j.cej.2023.143400

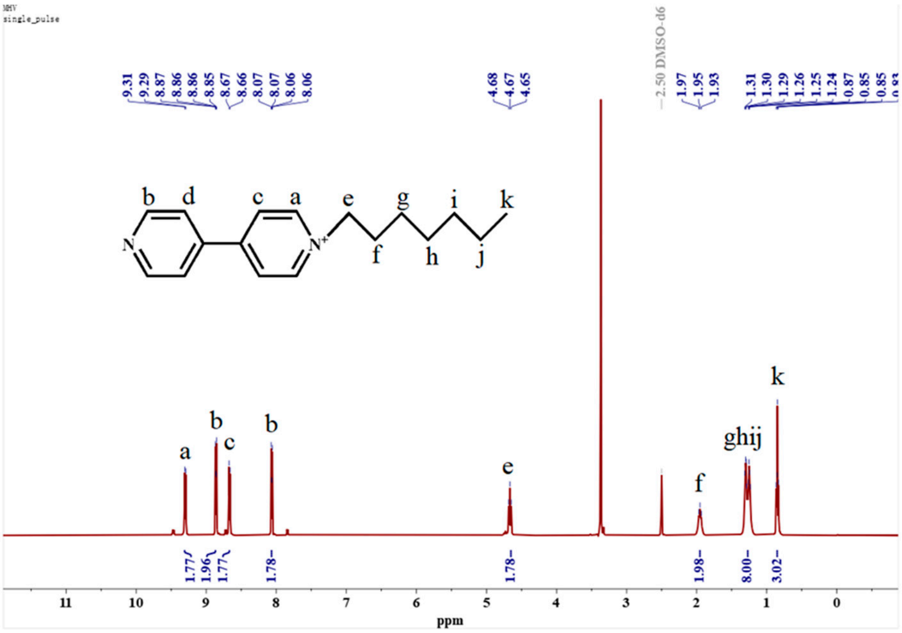

Figure.S3 1H-NMR of the Monoheptyl Viologen

Table. S2 The key simulation parameters for COMSOL Multiphysics simulation

Geometric dimensions	0.1*10*10mm、1*10*10mm
Dielectric constant	8.8(PVDF-HFP)、69(PC)
voltage	2V
Temperature	293K
Impedance	50 Ω

The steady-state equations used in the COMSOL Multiphysics AC/DC Module are given as follows：

$$\nabla \cdot D = \rho v$$

$$E = -\nabla V$$
